# Antitumor Effect of the Essential Oil from the Leaves of *Croton* *matourensis* Aubl. (Euphorbiaceae)

**DOI:** 10.3390/molecules23112974

**Published:** 2018-11-14

**Authors:** Emilly J. S. P. de Lima, Rafaela G. Alves, Gigliola M. A. D´Elia, Talita A. da Anunciação, Valdenizia R. Silva, Luciano de S. Santos, Milena B. P. Soares, Nállarett M. D. Cardozo, Emmanoel V. Costa, Felipe M. A. da Silva, Hector H. F. Koolen, Daniel P. Bezerra

**Affiliations:** 1 Metabolomics and Mass Spectrometry Research Group, Amazonas State University (UEA), Manaus, Amazonas, 690065-130, Brazil; emillyjulianasales@gmail.com (E.J.S.P.d.L.); gigliolamayara@gmail.com (G.M.A.D.); 2 Gonçalo Moniz Institute, Oswaldo Cruz Foundation (IGM-FIOCRUZ/BA), Salvador, Bahia, 40296-710, Brazil; rafalves09@gmail.com (R.G.A.); tali.andrade36@gmail.com (T.A.d.A.); valdeniziar@gmail.com (V.R.S.); luciano.biomed@gmail.com (L.d.S.S.); milena@bahia.fiocruz.br (M.B.P.S.); 3 Center of Biotechnology and Cell Therapy, Hospital São Rafael, Salvador, Bahia, 41253-190, Brazil; 4 Amazonia Museum (MUSA), Manaus, Amazonas, 69099-415, Brazil; nallarett.davila@gmail.com; 5 Department of Chemistry, Federal University of Amazonas (UFAM), Manaus, Amazonas, 69077-000, Brazil; emmanoelvc@gmail.com (E.V.C.) felipesaquarema@bol.com.br (F.M.A.d.S.)

**Keywords:** *Croton matourensis*, *Croton lanjouwensis*, antitumor, apoptosis, HepG2

## Abstract

*Croton matourensis* Aubl. (synonym *Croton lanjouwensis* Jabl.), popularly known as “orelha de burro”, “maravuvuia”, and/or “sangrad’água”, is a medicinal plant used in Brazilian folk medicine as a depurative and in the treatment of infections, fractures, and colds. In this work, we investigated the chemical composition and in vitro cytotoxic and in vivo antitumor effects of the essential oil (EO) from the leaves of *C. matourensis* collected from the Amazon rainforest. The EO was obtained by hydrodistillation using a Clevenger-type apparatus and characterized qualitatively and quantitatively by gas chromatography coupled to mass spectrometry (GC–MS) and gas chromatography with flame ionization detection (GC–FID), respectively. In vitro cytotoxicity of the EO was assessed in cancer cell lines (MCF-7, HCT116, HepG2, and HL-60) and the non-cancer cell line (MRC-5) using the Alamar blue assay. Furthermore, annexin V-FITC/PI staining and the cell cycle distribution were evaluated with EO-treated HepG2 cells by flow cytometry. In vivo efficacy of the EO (40 and 80 mg/kg/day) was demonstrated in C.B-17 severe combined **immunodeficient (SCID) mice with HepG2 cell xenografts. The EO included β-caryophyllene, thunbergol, cembrene, *p*-cymene, and β-elemene as major constituents. The EO exhibited promising cytotoxicity and was able to cause phosphatidylserine externalization and DNA fragmentation without loss of the cell membrane integrity in HepG2 cells. In vivo tumor mass inhibition rates of the EO were 34.6% to 55.9%. Altogether, these data indicate the anticancer potential effect of *C. matourensis*.

## 1. Introduction

The genus *Croton* (family Euphorbiaceae) contains approximately 1300 species that are found in tropical and subtropical regions of the world [1]. Plants from this genus have been used in the folk medicine to treat cancer, which include *C. palanostigma* Klotzsch [2,3], *C. lechleri* Müll. Arg. [4] and *C. tiglium* L. [5]. Moreover, numerous plants belonging to this genus have been reported with cytotoxic and antitumor potentials, including *C. palanostigma* Klotzsch [2], *C. regelianus* Müll. Arg. [6], *C. lechleri* Müll. Arg. [7], *C. urucurana* Baill. [8], *C. betulaster* Müll. Arg. [9], *C. tiglium* L. [10] and *C. crassifolius* Geiseler [11].

*Croton matourensis* Aubl. (synonym *Croton lanjouwensis* Jabl.) is a tree widely spread in the Amazon rainforest and in some regions of Central America (Panama). In Brazil, it is popularly known as “orelha de burro”, “maravuvuia”, and/or “sangrad’água”, and is used in folk medicine as a depurative and in the treatment of infections, fractures, and colds [12,13,14,15]. However, only few research papers are found for this species [16,17,18,19]. *α*-Pinene, elemecine, *α*-phellandrene, *p*-cymene, linalool, and *β*-caryophyllene were found as the main constituents in the essential oil (EO) from the leaves, fruit, inflorescence, and bark of *C. matourensis* collected from the Brazilian Amazon rainforest [16,17]. Additionally, the seco-labdane diterpene named maravuic acid was isolated from the bark of *C. matourensis* [18]. More recently, the chemical composition and cytotoxic activity of the EO from the leaves of *C. matourensis* collected in Venezuela were reported [19], in which fenchyl acetate, methyleugenol, isoelemicine, elemicine, spathulenol, and valencene were found as main constituents, and cytotoxic potential was observed in LoVo (human colon carcinoma) and HeLa (human cervical cancer) cell lines [19]. However, in vivo antitumor properties have not been investigated. In this work, we investigated the chemical composition, in vitro cytotoxicity, and in vivo antitumor effect of the EO obtained from the leaves of *C. matourensis* collected from the Amazon rainforest.

## 2. Results

### 2.1. Chemical Composition of the Essential Oil

The EO recovery from the leaves of *C. matourensis* was 0.34 ± 0.03% (*w*/*w*) and its chemical composition is presented in Table 1. The terpenoid class fully dominated the EO sample composition, in which, sesquiterpenes were the main representatives (46.53%), followed by diterpenes (26.94%), and monoterpenes (25.02%). Among them, 42 (representing 90.00% of the EO) were identified based on their retention indexes and mass spectral-fragments. The major compounds in the EO were β-caryophyllene (12.41 ± 1.02%), thunbergol (11.74 ± 1.11%), cembrene (7.12 ± 0.55%), *p*-cymene (5.05 ± 0.49%), and β-elemene (4.94 ± 0.35%).

### 2.2. In Vitro Cytotoxicity

In vitro cytotoxicity of the EO from the leaves of *C. matourensis* to human cancer cell lines MCF-7 (breast adenocarcinoma), HCT116 (colon carcinoma), HepG2 (hepatocellular carcinoma), HL-60 (promyelocytic leukemia), and human non-cancer cell line MRC-5 (lung fibroblasts) was assessed by the Alamar blue assay after 72 h of treatment. Table 2 presents the half maximal inhibitory concentrations (IC_50_) obtained. The EO displayed an IC_50_ value of 23.3 µg/mL for MCF-7, 28.9 µg/mL for HCT116, 28.5 µg/mL for HepG2, 17.8 µg/mL for HL-60, and 25.8 µg/mL for MRC-5. Doxorubicin was used as the positive control and showed an IC_50_ value of 0.3 µg/mL for MCF-7, 0.1 µg/mL for HCT116, 0.03 µg/mL for HepG2, 0.04 µg/mL for HL-60, and 0.2 µg/mL for MRC-5.

**Figure 1:**
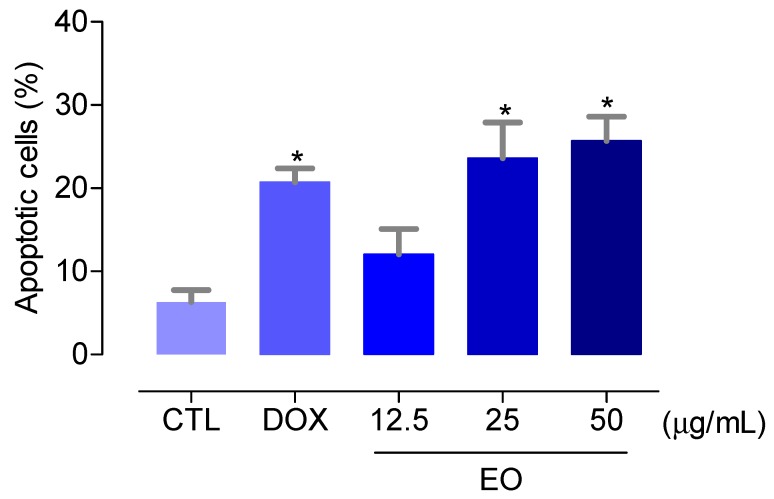
Effect of the essential oil (EO) from the leaves of *Croton matourensis* on the induction of apoptosis (early + late apoptotic cells) in HepG2 cells after 48 h of treatment, as determined by flow cytometry using annexin V-FITC/PI staining. The negative control (CTL) was treated with the vehicle (0.1% DMSO) used for diluting the EO. Doxorubicin (DOX, 1 µg/mL) was used as the positive control. Data are presented as the means ± SEM. of three independent experiments performed in duplicate. Ten thousand events were evaluated per experiment, and cellular debris was omitted from the analysis. * *P* < 0.05 compared with the negative control by ANOVA, followed by Bonferroni’s multiple comparison test.

Then, we quantified the cell viability using annexin V-FITC and PI double staining, and the cell fluorescence was measured by flow cytometry to determine the percentage of HepG2 cells in viable (cells that are annexin V-FITC/PI double-negative), early apoptotic (cells that are annexin V-FITC-positive and PI-negative), late apoptotic (cells that are annexin V-FITC/PI double-positive), and necrotic (cells that are annexin V-FITC-negative and PI-positive) stages after 48 h of treatment with the EO at concentrations of 12.5, 25, and 50 µg/mL, as shown in Figure 1. These concentrations were based at the IC_50_ value (28.5 µg/mL) of the EO for HepG2 cells, and since the doubling time of HepG2 cell is about 48 h, we used this time of treatment to evaluate the effect of the EO after one full cell cycle. The EO treatment caused an increase of the apoptotic (early + late apoptotic cells) cell death after 48 h of treatment (*P* < 0.05). No increase in the necrotic (annexin V-FITC-negative/PI-positive) cells was observed (*P* > 0.05). Doxorubicin also led to an increase of the apoptotic cells (*P* < 0.05). At the concentrations of 12.5, 25, and 50 μg/mL, the EO increased the apoptotic cell death to 12.1%, 23.6%, and 25.7%, against 6.3% observed at the control group. Doxorubicin, at 1 μg/mL, increased the apoptosis to 20.7%.

**Figure 2:**
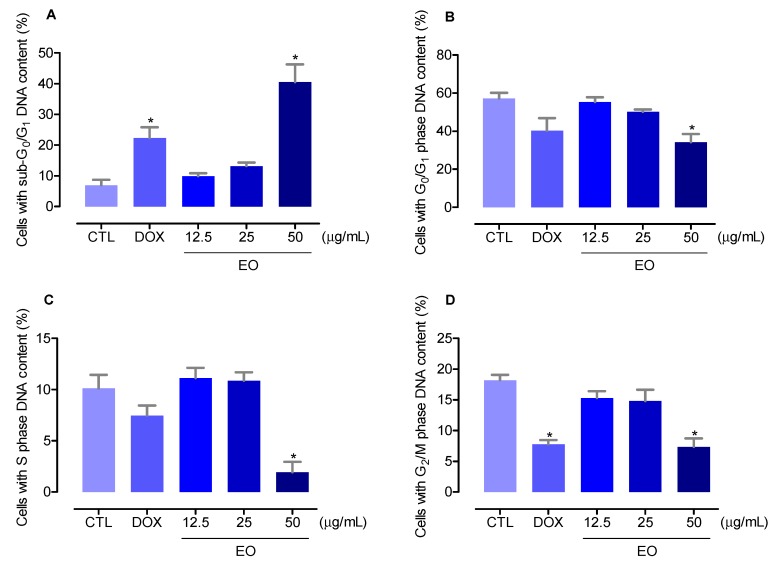
Effect of the essential oil (EO) from the leaves of *Croton matourensis* in the cell cycle distribution of HepG2 cells. (**A**) Percentage of cells with sub-G_0_/G_1_ DNA content (DNA fragmentation). (**B**) Percentage of cells with G_0_/G_1_ phase DNA content. (**C**) Percentage of cells with S phase DNA content. (**D**) Percentage of cells with G_2_/M phase DNA content. The negative control (CTL) was treated with the vehicle (0.1% DMSO) used for diluting the EO. Doxorubicin (DOX, 1 µg/mL) was used as the positive control. Data are presented as the means ± SEM. of three independent experiments performed in duplicate. Ten thousand events were evaluated per experiment, and cellular debris was omitted from the analysis. * *P* < 0.05 compared with the negative control by ANOVA, followed by Bonferroni’s multiple comparison test.

The cell cycle distribution in the EO-treated HepG2 cells was performed by the DNA content using flow cytometry after 48 h of treatment, as shown in Figure 2. All DNA that was sub-diploid in size (sub-G_0_/G_1_) was considered fragmented. EO-treated HepG2 cells presented an internucleosomal DNA fragmentation significantly increased (*P* < 0.05). Doxorubicin also significantly induced internucleosomal DNA fragmentation (*P* < 0.05). At the concentrations of 12.5, 25, and 50 μg/mL, the EO increased the DNA fragmentation to 10.0%, 13.2%, and 40.5%, against 7.0% observed at the control group. Doxorubicin, at 1 μg/mL, increased the DNA fragmentation to 22.4%. The cell cycle phases G_0_/G_1_, S, and G_2_/M were reduced proportionally.

### 2.3. In Vivo Antitumor Activity

**Figure 3:**
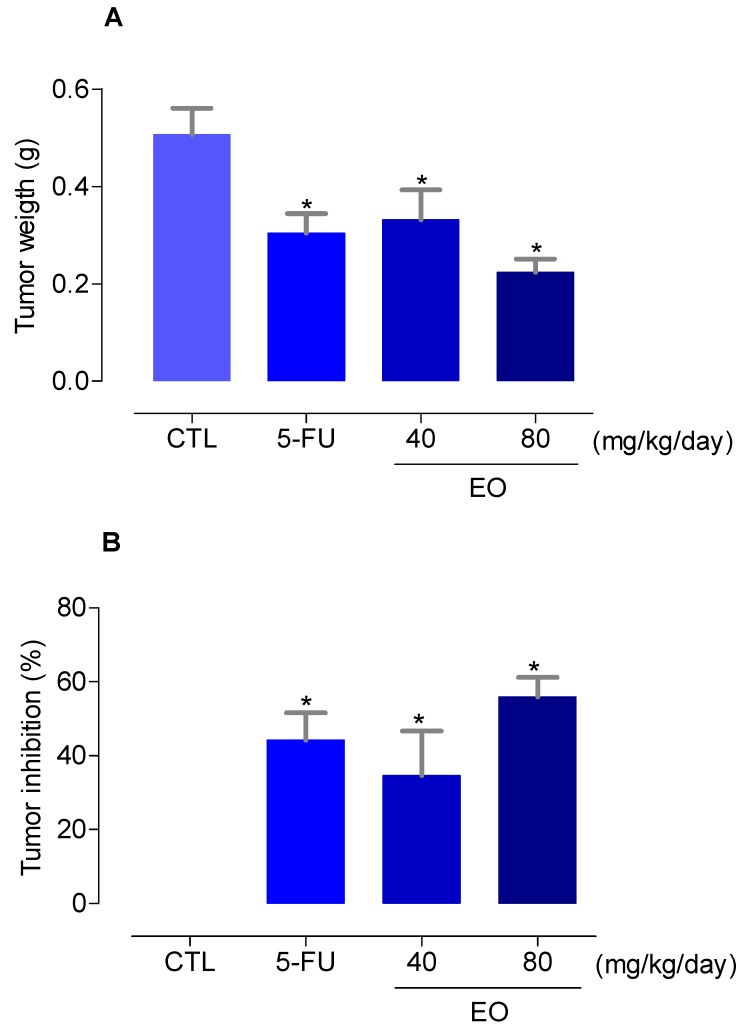
In vivo antitumor activity of the essential oil (EO) from the leaves of *Croton matourensis* in C.B-17 severe combined **immunodeficient (SCID) mice with HepG2 cell xenografts. (**A**) Tumor weight (g) after treatment. (**B**) Tumor inhibition (%) after treatment. Beginning 1 day after tumor implantation, the animals were treated through the intraperitoneal route for 21 consecutive days. The negative control (CTL) was treated with the vehicle (5% DMSO) used for diluting the EO. 5-Fluorouracil (5-FU, 10 mg/kg/day) was used as the positive control. Data are presented as means ± SEM. of 10–20 animals. * *P* < 0.05 compared to the negative control by ANOVA followed by Bonferroni’s multiple comparison test.

In vivo antitumor effect of the EO from the leaves of *C. matourensis* was assessed in C.B-17 severe combined **immunodeficient (SCID) mice with HepG2 cell xenografts. Animals were treated with the EO at doses of 40 and 80 mg/kg/day through intraperitoneal injections delivered once a day for 21 consecutive days. At the end of the treatment, the mean tumor mass weight of the negative control animals was 0.5 ± 0.1 g, as shown in Figure 3A. In the EO-treated animals, the mean tumor mass weights were 0.3 ± 0.1 g at 40 mg/kg/day dosage and 0.2 ± 0.03 g at 80 mg/kg/day dosage. Tumor mass inhibition rates of the EO were 34.6% to 55.9% (*P* < 0.05), as shown in Figure 3B. In this study, 5-fluorouracil at the dosage of 10 mg/kg/day was used as the positive control, which reduced the tumor weight by 44.2%.

In regard to the toxicological parameters investigated, no significant changes on body and organs (liver, kidney, lung, and heart) weight were seen on the EO-treated groups (*P* > 0.05), as shown in Table 3. Hematological parameters of the peripheral blood drawn from C.B-17 SCID mice with HepG2 cell xenografts were also analyzed, as shown in Table 4. Although some variations have been observed, no statistically significant alteration was found in the hematological parameters in the animals treated with the EO (*P* > 0.05).

## 3. Discussion

In this study, antitumor effect of the EO from the leaves of *C. matourensis* was reported for the first time at this communication. As mentioned above, a previous study investigated the chemical composition and cytotoxic activity of the EO from the leaves of *C. matourensis* collected in Venezuela [19]. These authors found fenchyl acetate, methyleugenol, isoelemicine, elemicine, spathulenol, and valencene as the main constituents of this EO*.* Moreover, previous studies with the EO from the leaves, fruit, inflorescence, and bark of *C. matourensis* collected from the Brazilian Amazon rainforest presented *α*-pinene, elemecine, α-phellandrene, *p*-cymene, linalool, and β-caryophyllene as the main constituents [16,17]. Herein, we observed a different chemical composition of the EO for *C. matourensis* in comparison with individuals collected in Venezuela, where β-caryophyllene, thunbergol, cembrene, *p*-cymene, and β-elemene were found as major constituents. Interestingly, when compared to with a specimen collected in central Amazonia, especially, in Brazil, a similar chemical composition is observed.

In the work of Compagnone et al. [19], the EO from the leaves of *C. matourensis* caused cytotoxicity in LoVo (human colon carcinoma) and HeLa (human cervical cancer) cell lines with IC_50_ values of 36.6 and 83.9 μg/mL, respectively, while inducing cytotoxicity in human dermis fibroblasts cells with an IC_50_ value of 132.7 μg/mL. In this work, we observed similar results where the EO was cytotoxic with IC_50_ values of 23.3 µg/mL for MCF-7, 28.9 µg/mL for HCT116, 28.5 µg/mL for HepG2, 17.8 µg/mL for HL-60, and 25.8 µg/mL for MRC-5.

In our cytotoxic screening program, EO with IC_50_ values below 30 μg/mL are considered promising for cancer drug development [20,21,22]. Therefore, the results obtained with the EO from the leaves of *C. matourensis* should be explored further. These data corroborate with previous results observed with EO obtained from *Croton* species. The EO from the leaves of *C. flavens* showed cytotoxicity in A-549 (human lung carcinoma) and DLD-1 (human colon adenocarcinoma) cells with IC_50_ values of 27 and 28 μg/mL, respectively [23]. The EO from the leaves of *C. regelianus* induced cytotoxic effect in HL-60, HCT-8 (human colon carcinoma), SF295 (human glioblastoma), MDA-MB-435 (human melanoma) cells, with IC_50_ values of 22.2, 40.0, 48.0, and 47.3 μg/mL, respectively [6]. The EO obtained from leaves of *C. campestres* exhibited cytotoxicity in MCF-7 and HT-29 (human colon adenocarcinoma) cells with IC_50_ values of 8.61 and 9.94 μg/mL, respectively [24].

Concerning the main constituents of the EO from the leaves of *C. matourensis,* some of them, e.g., β-caryophyllene, cembrene, *p*-cymene, and β-elemene, have been reported as cytotoxic agents [22,25,26,27,28], indicating that the mixture of them are associated with the cytotoxic and antitumor properties of this EO.

Phosphatidylserine externalization and DNA fragmentation, without loss of the cell membrane integrity, are cellular biochemical alterations that are often correlated with the apoptotic cell death [29]. Herein, we observed that the EO from the leaves of *C. matourensis* increased the phosphatidylserine exposure and internucleosomal DNA, without causing alterations in the cell membrane integrity (no increase in the PI-positive cells was observed). Moreover, the EO from the leaves of *Lippia gracilis, Guatteria blepharophylla*, and *Guatteria hispida* inhibited cell proliferation and induced cell death by apoptosis in HepG2 cells [25,30]. The EO from the aerial parts of *Salvia aurea*, *S. judaica,* and *S. viscosa* caused the apoptosis process and increasing reactive oxygen species in DU-145 (human prostate cancer) cells [31]. The EO from the leaves of *Pinus roxburghii* induced apoptosis along with inhibition of NF-*κ*B and inhibited the expression of genes associated to cell survival (survivin, c-FLIP, Bcl-2, Bcl-xL, c-Myc, c-IAP2), proliferation (cyclin D1), and metastasis (MMP-9) in KBM-5 (chronic myeloid leukemia) cells [32].

In addition, we demonstrated that the EO from the leaves of *C. matourensis* has in vivo antitumor effect in C.B-17 SCID mice with HepG2 cell xenografts. The antitumor effect of the EO from the leaves of *C. regelianus* has been previously reported using sarcoma 180 as tumor model, while the EO inhibited the tumor development by 28.1% and 31.8%, at doses of 50 and 100 mg/kg/day, and the ascaridole, one of its main constituents, inhibited the tumor development by 33.9% and 33.3%, at doses of 10 and 20 mg/kg/day, respectively [6]. Herein, the EO from the leaves of *C. matourensis* inhibited the tumor mass by 34.6% to 55.9% at doses of 40 and 80 mg/kg/day, respectively, and although the EO was less potent than the positive control 5-fluorouracil, the EO presented similar or superior efficacy to that observed for it. The positive control 5-fluorouracil inhibited the tumor mass by 44.2% at a dose of 10 mg/kg/day.

In conclusion, the EO from the leaves of *C. matourensis* has β-caryophyllene, thunbergol, cembrene, *p*-cymene, and β-elemene as major constituents. Moreover, the EO has an in vitro and in vivo anti-liver cancer effect. These data indicate the anticancer potential effect of this plant.

## 4. Material and Methods

### 4.1. Botanical Material

Leaves from the species *C. matourensis* were collected in March 2014 in the green area of the Adolpho Ducke botanical garden at the Amazonia Museum (MUSA) (3°01′12.8″ S, 59°56′23.2″ W) from a specimen previously identified by N.M.D. Cardozo and catalogued (#492).

### 4.2. Essential Oil Extraction

After collection, the aerial parts were directly extracted by hydrodistillation in a Clevenger-type apparatus. For this, 300 g of fresh and crushed material was extracted for a period of 4 h in 1000 mL of ultrapure water (18.2 MΩ). Then, the obtained oils were extracted with CH_2_Cl_2_, dried over anhydrous Na_2_SO_4_ and filtered through a nylon membrane (pore size 0.22 μm, Whatman, Maidstone, UK). The resulting EO were weighed in vials and stored at −4 °C prior to chemical analysis.

### 4.3. Chemical Analysis

The qualitative analysis was performed by gas chromatography coupled to mass spectrometry (GC-MS) with an equipment model GCMS/QP2010 Plus (Shimadzu, Kyoto, Japan) using a selective detector and a capillary column Rtx-5 MS (30 m × 0.25 mm × 0.25, Restek). Helium was used as carrier gas with a flow of 1.02 mL/min. Injections (1 μL) were performed with EO solutions at 2 mg/mL in CH_2_Cl_2_ using a split ratio of 1:50. The column temperature program was 60 to 280 °C with gradual increase of 3 °C/min. The temperatures of the injector and the ion source were 220 °C and 260 °C, respectively. Preliminary identifications of the constituents were performed based on comparison of experimental spectra with those stored in the Wiley 8th edition library (similarities > 90%). The confirmation of the identifications was performed by the calculation of the retention indexes (RI) according to the Van den Dool and Kratz equation [33] in comparison to a homologous series of linear hydrocarbons (C7-C30). A semi-quantitative analysis was performed to obtain the relative amount of each component of the EO. For this procedure, a gas chromatography with flame ionization detection (GC-FID) system, model GC2010 (Shimadzu) equipped with a Rtx-5 capillary column, was used. The same conditions of the GC-MS analysis were employed to ensure reproducibility. Relative amounts (%) were calculated in relation to the total area of the chromatogram.

### 4.4. In Vitro Assays

#### 4.4.1. Cells

Human cancer cell lines MCF-7 (breast adenocarcinoma), HCT116 (colon carcinoma), HepG2 (hepatocellular carcinoma), HL-60 (promyelocytic leukemia), and human non-cancer cell line MRC-5 (lung fibroblast) were obtained from the American Type Culture Collection (ATCC, Manassas, VA, USA). The cells were cultured as recommended by the ATCC. Cell viability was examined via trypan blue exclusion method for all experiments.

#### 4.4.2. Cell Viability Assay

Cell viability was measured by the Alamar blue assay and was performed as previously described [34,35,36]. For all experiments, cells were plated in 96-well plates. The EO was dissolved in dimethyl sulfoxide (DMSO, Vetec Química Fina Ltd., Duque de Caxias, RJ, Brazil) and added to each well and incubated for 72 h. Doxorubicin (purity ≥ 95%, doxorubicin hydrochloride, Laboratory IMA S.A.I.C., Buenos Aires, Argentina) was used as the positive control. At the end of the treatment, 20 μL of a stock solution (0.312 mg/mL) of resazurin (Sigma-Aldrich Co., Saint Louis, MO, USA) was added to each well. Absorbances at 570 and 600 nm were measured using a SpectraMax 190 Microplate Reader (Molecular Devices, Sunnyvale, CA, USA).

#### 4.4.3. Annexin-V/PI Staining Assay

For apoptosis quantification, FITC Annexin V Apoptosis Detection Kit I (BD Biosciences, San Jose, CA, USA) was used, and the analysis performed according to the manufacturer’s instructions. Cell fluorescence was measured by flow cytometry with a BD LSRFortessa cytometer. For flow cytometry analyses, 10^4^ events were recorded per sample with the BD FACSDiva Software (BD Biosciences) and Flowjo Software 10 (Flowjo LLC, Ashland, OR, USA). Cellular debris was omitted from the analysis.

#### 4.4.4. Internucleosomal DNA Fragmentation and Cell Cycle Distribution

The internucleosomal DNA fragmentation and cell cycle distributions were determined using propidium iodide (PI) (Sigma-Aldrich Co. St. Louis, MO, USA) in permeabilized cells, and performed as previously described [37]. Cell fluorescences were measured by flow cytometry as described above.

### 4.5. In Vivo Assays

#### 4.5.1. Animals

A total of 50 C.B*-*17 severe combined **immunodeficient (SCID) mice (females, 25–30 g) were obtained and maintained at Gonçalo Moniz Institute-FIOCRUZ animal facilities (Salvador, Bahia, Brazil). Animals were housed in cages with free access to food and water. All animals were subjected to a 12:12 h light-dark cycle (lights on at 6:00 a.m.). A local animal ethics committee approved the experimental protocol employed (number #06/2015).

#### 4.5.2. Human Hepatocellular Carcinoma Xenograft Model

HepG2 cells (10^7^ cells per 500 µL) were implanted subcutaneously into the left front armpits of the mice. At the beginning of the experiment, mice were randomly divided into four groups. Group 1: animals received injections of vehicle with 5% DMSO solution (*n* = 20). Group 2: animals received injections of 5-fluorouracil (10 mg/kg/day, Sigma-Aldrich, *n* = 10). Group 3: animals received injections of the EO at 40 mg/kg/day (*n* = 10). Group 4: animals received injections of the EO at 80 mg/kg/day (*n* = 10). These dosages were selected based on previous works using EO in tumor models in mice [25,38]. Beginning 1 day after tumor implantation, the animals were treated through the intraperitoneal route for 21 consecutive days. One day after the end of the treatment, animals were anesthetized, and peripheral blood samples were collected from the brachial artery. Animals were euthanized by anesthetic overdose, and tumors were excised and weighed.

#### 4.5.3. Toxicological Evaluation

The mice were weighed at the beginning and end of the experiment to evaluate the toxicological effects. Animals were observed for signs of abnormality throughout the study. A hematological analysis was performed using the Advia 60 hematology system (Bayer, Leverkusen, Germany). Livers, kidneys, lungs, and hearts were removed, weighed, and examined for signs of gross lesion formation, color change, and/or hemorrhaging.

### 4.6. Statistical Analysis

Data were presented as means ± standard error of the mean (SEM) or as IC_50_ values with 95% confidence intervals obtained by nonlinear regressions. Differences among the experimental groups were compared through analysis of variance (ANOVA) followed by Bonferroni’s multiple comparison test (*P* < 0.05). All statistical analyses were performed using GraphPad Prism (Intuitive Software for Science; San Diego, CA, USA).

## Figures and Tables

**Table 1:** Chemical composition of the essential oil from the leaves of *Croton matourensis.*

Number	Compound	Retention Time (min)	RI Theo. ^a^	RI Exp. ^b^	Proportion Area (%) ^c^
1	α-thujene	5.28	924	924	1.99 ± 0.21
2	α-pinene	5.48	932	931	3.41 ± 0.34
3	sabinene	6.60	969	969	0.11 ± 0.03
4	β-pinene	6.72	974	974	0.25 ± 0.04
5	β-myrcene	7.11	988	987	0.26 ± 0.04
6	α-phellandrene	7.59	1002	1002	3.90 ± 0.28
7	α-terpinene	8.02	1014	1014	0.54 ± 0.30
8	*p*-cymene	8.31	1020	1020	5.05 ± 0.49
9	limonene	8.46	1024	1023	1.85 ± 0.18
10	eucalyptol	8.57	1026	1026	0.15 ± 0.02
11	β-ocimene	9.18	1032	1032	0.13 ± 0.02
12	γ-terpinene	9.62	1054	1054	2.02 ± 0.23
13	terpinolene	10.85	1086	1086	2.15 ± 0.18
14	linalool	11.30	1095	1095	3.85 ± 0.34
15	4-terpineol	14.87	1130	1130	0.25 ± 0.05
16	α-terpineol	15.52	1131	1131	0.21 ± 0.04
17	α-copaene	24.62	1374	1373	1.92 ± 0.15
18	α-bourbonene	25.06	1387	1387	0.36 ± 0.05
19	β-elemene	25.48	1389	1388	4.94 ± 0.35
20	β-caryophyllene	26.74	1417	1417	12.41 ± 1.02
21	α-humulene	28.36	1436	1436	2.52 ± 0.20
22	β-farnesene	28.62	1440	1440	2.33 ± 0.14
23	aromadendrane	28.72	1460	1559	0.64 ± 0.11
24	germacrene B	29.52	1480	1480	1.07 ± 0.29
25	α-amorphene	29.71	1483	1483	0.90 ± 0.13
26	α-selinene	30.37	1498	1498	0.95 ± 0.08
27	β-bisabolene	31.06	1505	1505	0.59 ± 0.05
28	γ-cadinene	31.74	1513	1513	0.99 ± 0.11
29	α-elemol	32.91	1548	1548	0.96 ± 0.09
30	spathulenol	34.18	1577	1577	1.29 ± 0.15
31	caryophyllene oxide	34.41	1582	1582	3.19 ± 0.31
32	globulol	34.81	1590	1590	0.65 ± 0.12
33	viridiflorol	35.33	1592	1592	0.24 ± 0.07
34	salvial-4(14)-en-1-one	35.60	1593	1593	0.34 ± 0.09
35	α-cadinol	37.30	1638	1638	0.38 ± 0.08
36	τ-muurolol	37.41	1643	1643	0.48 ± 0.10
37	β-eudesmol	37.55	1649	1649	0.52 ± 0.13
38	α-eudesmol	37.61	1652	1652	0.48 ± 0.11
39	bulnesol	48.93	1670	1670	4.37 ± 0.48
40	cembrene	53.40	1937	1938	7.12 ± 0.55
41	thunbergol	56.69	2061	2063	11.74 ± 1.11
42	geranyllinalool	56.90	2125	2125	2.30 ± 0.34
_Σtotal identified_					90.00%

^a^ Retention indexes (RI) calculated with the Van den Dool and Kratz equation, ^b^ Main observed fragments and molecular mass (MM), ^c^ Proportional area relative to the total area of chromatogram after eliminating peaks arising of contamination and/or column bleeding.

**Table 2:** Half maximal inhibitory concentration (IC_50_) values of the cytotoxic activity of the essential oil (EO) from the leaves of *Croton matourensis.*

Cell Lines	Origin	Histological Type	IC_50_ in µg/mL	
			EO	DOX
**Cancer cells**				
MCF-7	Human	Breast adenocarcinoma	23.3 18.2–29.7	0.3 0.2–0.4
HCT116	Human	Colon carcinoma	28.9 22.1–37.8	0.1 0.1–0.2
HepG2	Human	Hepatocellular carcinoma	28.5 17.1–37.3	0.03 0.01–0.2
HL-60	Human	Promyelocytic leukemia	17.8 14.6–21.7	0.04 0.02–0.08
**Non-cancer cell**				
MRC-5	Human	Lung fibroblast	25.8 22.0–30.4	0.2 0.1–0.5

Data are presented as IC_50_ values, in μg/mL, with respective 95% confidence interval obtained by nonlinear regression from at least three independent experiments performed in duplicate, measured by Alamar blue assay after 72 h of treatment. Doxorubicin (DOX) was used as the positive control.

**Table 3:** Effect of the essential oil (EO) from the leaves of *Croton matourensis* on body and relative organ weight from C.B-17 SCID mice with HepG2 cell xenografts.

Parameters	CTL	5-FU	EO	
Dose (mg/kg/day)	-	10	40	80
Initial body weight (g)	21.4 ± 0.5	19.6 ± 0.6	22.0 ± 0.5	19.9 ± 0.6
Final body weight (g)	22.1 ± 0.5	20.5 ± 0.5	21.0 ± 0.3	20.9 ± 0.4
Liver (g/100 g body weight)	4.8 ± 0.2	4.8 ± 0.2	5.0 ± 0.3	5.2 ± 0.3
Kidney (g/100 g body weight)	1.5 ± 0.1	1.5 ± 0.1	1.4 ± 0.1	1.5 ± 0.1
Heart (g/100 g body weight)	0.5 ± 0.1	0.6 ± 0.1	0.6 ± 0.1	0.5 ± 0.1
Lung (g/100 g body weight)	0.8 ± 0.1	0.8 ± 0.1	0.8 ± 0.1	0.7 ± 0.1

Beginning 1 day after tumor implantation, the animals were treated through the intraperitoneal route for 21 consecutive days. The negative control (CTL) was treated with the vehicle (5% DMSO) used for diluting the EO. 5-Fluorouracil (5-FU, 10 mg/kg/day) was used as the positive control. Data are presented as means ± SEM. of 10–20 animals.

**Table 4:** Effect of the essential oil (EO) from the leaves of *Croton matourensis* on hematological parameters of peripheral blood from C.B-17 SCID mice with HepG2 cell xenografts.

Parameters	CTL	5-FU	EO	
Dose (mg/kg/day)	-	10	40	80
Erythrocytes (10^6^/mm^3^)	5.2 ± 1.1	7.6 ± 0.8	4.9 ± 1.2	7.6 ± 1.1
Hemoglobin (g/dL)	21.2 ± 4.8	17.7 ± 3.1	26.9 ± 0.7	17.7 ± 2.2
Hematocrit (%)	22.0 ± 4.7	38.9 ± 0.3	9.5 ± 0.3	40.1 ± 1.4
MCV (fL)	43.8 ± 0.4	45.0 ± 3.0	42.0 ± 0.0	45.3 ± 0.5
Platelets (10^3^/mm^3^)	247.2 ± 38.5	222.1 ± 41.6	519.3 ± 136.6	279.7 ± 37.2
Leukocytes (10^3^/mm^3^)	5.2 ± 0.8	2.5 ± 0.6	7.1 ± 0.6	3.5 ± 0.5
Differential leukocytes (%)				
Granulocytes	24.1	28.4	26.8	26.7
Lymphocytes	41.5	46.1	45.4	51.7
Monocytes	33.6	25.5	27.8	21.7

Beginning 1 day after tumor implantation, the animals were treated through the intraperitoneal route for 21 consecutive days. The negative control (CTL) was treated with the vehicle (5% DMSO) used for diluting the EO. 5-Fluorouracil (5-FU, 10 mg/kg/day) was used as the positive control. Data are presented as means ± SEM. of 7–14 animals. MCV: Mean corpuscular volume.
